# Supplementary material for: Comparison of Direct Oral Anticoagulant Use for the Treatment of Non-Valvular Atrial Fibrillation in Pivotal Clinical Trials vs. the Real-World Setting: A Population-Based Study from Southern Italy
Source: Pharmaceuticals (Basel). 2021 Mar 25;14(4):290. doi: 10.3390/ph14040290 (PMC8064382; doi:10.3390/ph14040290)
Supplement: Supplementary file 1 [file pharmaceuticals-14-00290-s001.zip › Supplementary Methods.pdf]

Supplementary Methods

Details about RCT data simulations:

Data of all RCTs were simulated and 1:1 matched with a real world population dataset (i.e. Palermo Claims database), following these steps:

1) For each specific RCT and for each treatment arm, data were simulated on the basis of the reported summary statistics (i.e. number of subjects in each arm, age, sex, presence of stroke/TIA, heart failure, diabetes, hypertension and CHADS2 score) and inclusion criteria.

2) It was assumed that the values of each simulated variable were extracted from marginal distributions rather than conditional or joint distributions (so that each variable can be considered statistically independent from the others).

For instance, consider the “Dabigatran 110 mg” treatment arm (RELY trial). 6,015 records were simulated from the following random variables:

Age ~ Normal (mean=71.4, standard deviation=8.6)
Sex (males subjects) ~ Binomial (p=3,865/6,015=0.64256)
Presence of Stroke/TIA ~ Binomial (p=1,195/6,015=0.19867)
Presence of Heart Failure ~ Binomial (p=1,937/6,015=0.322028)
Presence of Diabetes ~ Binomial (p=1,409/6,015=0.234248)
Presence of Hypertension ~ Binomial (p=4,738/6,015=0.787697)

CHADS2 score was straightforwardly calculated on the basis of the simulated comorbidities, according to the following formula: 1*(Age≥75) + 2*(Stroke/TIA=”Yes”) + 1*(Heart Failure=”Yes”) + 1*(Diabetes=”Yes”) + 1*(Hypertension=”Yes”)

3) Data from the simulated RCT and the specific treatment arm were appended with data from Palermo Claims database.

4) Individual propensity score (i.e. the individual probability of being selected in the simulated dataset) was estimated and 1:1 matching was performed, using Parsons’ greedy 5 to 1 digit match algorithm.

5) Efficacy and safety outcomes were assessed in the matched real world population data.

6) Simulations were re-run (step 1) 1,000 times for the same scenario (i.e. RCT and treatment arm combination) and 95% confidence interval of the empirical distribution of each outcome’s proportion was calculated.

7) Change the scenario (i.e. RCT and new treatment arm combination) and re-run simulations (step 1).
