# Supplementary material for: Comparison of Direct Oral Anticoagulant Use for the Treatment of Non-Valvular Atrial Fibrillation in Pivotal Clinical Trials vs. the Real-World Setting: A Population-Based Study from Southern Italy
Source: Pharmaceuticals (Basel). 2021 Mar 25;14(4):290. doi: 10.3390/ph14040290 (PMC8064382; doi:10.3390/ph14040290)
Supplement: Supplementary file 1 [file pharmaceuticals-14-00290-s001.zip › Table S1.pdf]

Supplement Table S1. Comparative characteristics of DOACs pivotal trials and Palermo claims database.

	**DABIGATRAN**		**RIVAROXABAN**		**APIXABAN**		**EDOXABAN**	
**Data source**	**RE-LY (2009)**	**Palermo claims database**	**ROCKET- AF (2011)**	**Palermo claims database**	**ARISTOTLE (2011)**	**Palermo claims database**	**ENGAGE AF-TIMI 48 (2013)**	**Palermo claims database**
**Study design**	Pivotal randomized trial	Observational, non-interventional, retrospective study	Pivotal, multicenter, randomized, double-blind, double-dummy, event-driven trial	Observational, non-interventional, retrospective study	Pivotal, randomized, double-blind trial	Observational, non-interventional, retrospective study	Pivotal, randomized, double-blind, double-dummy trial	Observational, non-interventional, retrospective study
**Study Years**	Enrolment: December 2005-December 2007;<br>Final follow-up visit: April 2009	January 2013-December 2017	Enrolment: December 2006- June 2009<br>Final follow-up visit: September 2010	January 2013-December 2017	Enrolment: December 2006- April2010<br>Final follow-up visit: May 2011	January 2013-December 2017	Enrolment: November 2008- November 2010<br>Final follow-up visit: May 2013	January 2013-December 2017
**Study drugs**	Dose-specific comparison		Non dose-specific comparison		Non dose-specific comparison		Dose-specific comparison	
**Study Population**	N=6,015<br>N=6,076	N= 794<br>N=635	N=7,131	N= 3,028	N=9,120	N= 2,113	N=7,034<br>N=7,035	N= 253<br>N= 269
	From: USA, Canada, Central Europe, Western Europe, Latin America, Asia and other	From: Palermo LHU	From: North America, Latin America, West Europe, East Europe, Asia Pacific	From: Palermo LHU	From: North America, South America, Europe or Asia Pacific	From: Palermo LHU	From: North America, Latin America, Western Europe, Eastern Europe, Asia pacific region and South Africa	From: Palermo LHU
