# Supplementary material for: Comparison of Direct Oral Anticoagulant Use for the Treatment of Non-Valvular Atrial Fibrillation in Pivotal Clinical Trials vs. the Real-World Setting: A Population-Based Study from Southern Italy
Source: Pharmaceuticals (Basel). 2021 Mar 25;14(4):290. doi: 10.3390/ph14040290 (PMC8064382; doi:10.3390/ph14040290)
Supplement: Supplementary file 1 [file pharmaceuticals-14-00290-s001.zip › Table S2.pdf]

Supplement Table S2. List of study drugs.

DOAC	ATC code	AIC code
**Dabigatran 110 mg**	B01AE07	038451050
		038451062
		038451074
		038451086
		038451148
**Dabigatran 150 mg**		038451124
		038451098
		038451100
		038451112
		038451136
**Rivaroxaban 10 – 15 – 20 mg**	B01AF01	-
**Apixaban 2.5 – 5 mg**	B01AF02	-
**Edoxaban 30 mg**	B01AF03	044315024
		044315137
		044315125
		044315152
		044315048
		044315051
		044315063
		044315149
		044315075
		044315087
		044315099
		044315101
		044315113
**Edoxaban 60 mg**		044315036
		044315265
		044315253
		044315289
		044315176
		044315188
		044315190
		044315277
		044315202
		044315214
		044315226
		044315238
		044315240

Legend: DOAC= direct oral anticoagulant; ATC= Anatomical Therapeutic Chemical classification system; AIC= Italian marketing authorization.
