# Supplementary material for: Comparison of Direct Oral Anticoagulant Use for the Treatment of Non-Valvular Atrial Fibrillation in Pivotal Clinical Trials vs. the Real-World Setting: A Population-Based Study from Southern Italy
Source: Pharmaceuticals (Basel). 2021 Mar 25;14(4):290. doi: 10.3390/ph14040290 (PMC8064382; doi:10.3390/ph14040290)
Supplement: Supplementary file 1 [file pharmaceuticals-14-00290-s001.zip › Table S3.pdf]

Supplement Table S3. ICD-9 codes identifying effectiveness outcome.

Category	ICD9	Description
Ischemic Stroke	433	Occlusion and stenosis of precerebral arteries
	434	Occlusion of cerebral arteries
	433.0	Occlusion and stenosis of basilar artery
	433.1	Occlusion and stenosis of carotid artery
	433.2	Vertebral artery
	433.3	Multiple and bilateral
	433.8	Other specified precerebral artery
	433.9	Unspecified precerebral artery
	434.0	Cerebral thrombosis
	434.1	Cerebral embolism
	434.9	Cerebral artery occlusion unspecified
	437.1	Other generalized ischemic cerebrovascular disease
	433.01	Occlusion and stenosis of basilar artery with cerebral infarction
	433.11	Occlusion and stenosis of carotid artery with cerebral infarction
	433.21	Occlusion and stenosis of vertebral artery with cerebral infarction
	433.31	Occlusion and stenosis of multiple and bilateral precerebral arteries with cerebral infarction
	433.81	Occlusion and stenosis of other specified precerebral artery with cerebral infarction
	433.91	Occlusion and stenosis of unspecified precerebral artery with cerebral infarction
	434.01	Cerebral thrombosis with cerebral infarction
	434.11	Cerebral embolism with cerebral infarction
	434.91	Cerebral artery occlusion, unspecified with cerebral infarction
