# Supplementary material for: Comparison of Direct Oral Anticoagulant Use for the Treatment of Non-Valvular Atrial Fibrillation in Pivotal Clinical Trials vs. the Real-World Setting: A Population-Based Study from Southern Italy
Source: Pharmaceuticals (Basel). 2021 Mar 25;14(4):290. doi: 10.3390/ph14040290 (PMC8064382; doi:10.3390/ph14040290)
Supplement: Supplementary file 1 [file pharmaceuticals-14-00290-s001.zip › Table S4.pdf]

Supplement Table S4. ICD-9 codes identifying safety outcomes.

Category	ICD9	Description
Gastrointestinal bleeding	455.2	Internal hemorrhoids with other complication
	455.5	External hemorrhoids with other complication
	455.8	Unspecified hemorrhoids with other complication
	456.0	Esophageal varices with bleeding
	530.7	Gastroesophageal laceration-hemorrhage syndrom
	456.20	Esophageal varices in diseases classified elsewhere: with bleeding
	535.61	Duodenitis with hemorrhage
	535.71	Eosinophilic gastritis with hemorrhage
	456.0	Esophageal varices with bleeding
	456.20	Esophageal varices in diseases classified elsewhere with bleeding
	530.21	Ulcer of esophagus with bleeding
	530.82	Esophageal hemorrhage
	531.0	Gastric ulcer acute with hemorrhage
	531.2	Gastric ulcer acute with hemorrhage and perforation
	531.4	Gastric ulcer chronic or unspecified with hemorrhage
	531.6	Gastric ulcer chronic or unspecified with hemorrhage and perforation
	532.0	duodenal ulcer acute with hemorrhage
	532.2	Duodenal ulcer acute with hemorrhage and perforation
	532.4	duodenal ulcer chronic or unspecified with hemorrhage
	532.6	Duodenal ulcer chronic or unspecified with hemorrhage and perforation
	533.0	Peptic ulcer, site unspecified acute with hemorrhage
	533.2	Peptic ulcer, site unspecified acute with hemorrhage and perforation
	533.4	Peptic ulcer, site unspecified chronic or unspecified with hemorrhage
	533.6	Peptic ulcer, site unspecified chronic or unspecified with hemorrhage and perforati
	534.0	Gastrojejunal ulcer acute with hemorrhage
	534.2	Gastrojejunal ulcer acute with hemorrhage and perforation
	534.4	Gastrojejunal ulcer chronic or unspecified with hemorrhage
	534.6	Gastrojejunal ulcer chronic or unspecified with hemorrhage and perforation
	535.01	Acute gastritis, with hemorrhage
	535.11	Atrophic gastritis with hemorrhage
	535.21	Gastric mucosal hypertrophy with hemorrhage
	535.31	Alcoholic gastritis with hemorrhage
	535.41	Other specified gastritis, with hemorrhage
	535.51	Unspecified gastritis and gastroduodenitis, with hemorrhage
	537.83	Angiodysplasia of stomach and duodenum with hemorrhage
	557.0	Acute vascular insufficiency of intestine
	562.02	Diverticulosis of small intestine with hemorrhage
	562.03	Diverticulitis of small intestine with hemorrhage
	562.12	Diverticulosis of colon with hemorrhage
	562.13	Diverticulitis of colon with hemorrhage
	569.3	Hemorrhage of rectum and anus

	569.85	Angiodysplasia of intestine with hemorrhage
	578	Gastrointestinal hemorrhage
Intracranial bleeding	853.0	Other and unspecified intracranial hemorrhage following injury Without mention of open intracranial wound
	430	Subarachnoid hemorrhage
	431	Intracerebral hemorrhage
	432	Other and unspecified intracranial hemorrhage
	852	Subarachnoid, subdural, and extradural hemorrhage, following injury
	853	Other and unspecified intracranial hemorrhage following injury
